# microRNA-802/Rnd3 pathway imposes on carcinogenesis and metastasis of fine particulate matter exposure

**DOI:** 10.18632/oncotarget.9019

**Published:** 2016-04-26

**Authors:** Xiaobo Li, Yang lv, Na Gao, Hao Sun, Runze Lu, Hongbao Yang, Chengcheng Zhang, Qingtao Meng, Shenshen Wu, Ai-Qun Li, Yankai Xia, Rui Chen

**Affiliations:** ^1^ Key Laboratory of Environmental Medicine Engineering, Ministry of Education, School of Public Health, Southeast University, Nanjing 210009, China; ^2^ Department of Histology and Embryology, Hebei North University, Zhangjiakou, 075000, China; ^3^ Center for Drug Safety Evaluation and Research, China Pharmaceutical University, Nanjing, 211198, China; ^4^ School of Public Health, Medical College, Wuhan University of Science and Technology, Wuhan, 430081, China; ^5^ Key Laboratory of Reproductive Medicine, Institute of Toxicology, Nanjing Medical University, Nanjing 210009, China; ^6^ State Key Laboratory of Bioelectronics, Southeast University, Nanjing, 210096, China

**Keywords:** PM_2.5_, microRNA, lung cancer, carcinogenesis, actin

## Abstract

Recent studies have linked ambient fine particulate matter (PM_2.5_) to increased lung cancer mortality and morbidity. However, the underlying mechanism causing the adverse effects of PM_2.5_ is less clear. In the present study, post-transcriptional profiling was used to explore biological pathways involved in PM_2.5_-induced pulmonary disorders. The carcinogenesis and metastasis of PM_2.5_ exposure were evaluated by long-term PM_2.5_ exposure tests. We observed dysregulation of actin in A549 cells line and dysplasia in the lungs of mice exposed to PM_2.5_. Both PM_2.5_-exposed cells and animals showed increased Rnd3 expression levels. Moreover, miR-802 mimics rescued actin disorganization *in vitro* and alveolitis *in vivo*. Long-term exposure to PM_2.5_ promoted carcinogenesis and metastasis of pulmonary cells. Decreased miR-802 expression levels in the serum samples of PM_2.5_-treated mice and individuals from moderately polluted cities were observed. Increased Rnd3 expression levels in lung cancers tissues have been identified by a genome database TCGA, and have been linked to less overall survival probabilities of lung cancer patients. Our findings suggest that dysregulation of actin cytoskeleton and down-regulation of miR-802 expression might be the underlying mechanism involved in the adverse effects of PM_2.5_ exposure. In addition, long-term exposure to PM_2.5_ demonstrated strong associations with malignant pulmonary disorders.

## INTRODUCTION

Previous studies have highlighted the relationship of PM_2.5_ exposure to the exacerbation of asthma, bronchitis, chronic obstructive pulmonary disease (COPD), and lung cancer [1-4]. In 2013, the International Agency for Research on Cancer classified outdoor air pollution and particulate matter (PM) as carcinogens to humans [1]. Population based studies strengthen the evidence that PM_2.5_ are correlated to increases in lung cancer mortality [5-7]. Although some biological mechanisms have been explored, including inflammation, oxidative stress, and epigenetic modification [8, 9], the mechanisms underlying PM_2.5_-induced pulmonary toxicity are not completely understood.

MicroRNAs (miRNAs) are endogenous small noncoding RNA molecules, 20 to 23 nucleotides in length, which are thought to regulate approximately 30% of all human genes. Therefore, the dysregulation of miRNAs is involved in many pathological processes [10]. Several studies have investigated changes in miRNAs expression in response to PM_2.5_ or its major components. Jardim et al. [11] first reported that diesel exhaust particles induced dysregulation of miRNAs expression in human airway cells, which may be related to tumorigenesis. In addition, PM, carbon black particles, and metal-rich particles reportedly induced altered miRNAs exposure *in vitro*, in animal models and human leukocytes [12-15].

miRNAs reduce their target gene expression through degradation of mRNA or repression of its translation [16]. Integrative analysis of miRNAs and their target mRNA expression using bioinformatics tools is thought to be useful for understanding the mechanisms underlying miRNA involvement in various human diseases [17]. In terms of accuracy, quantitative proteomic profiling is considered more reliable and comprehensive for predicting miRNA targets than traditional mRNA microarray analysis [18]. Omics analysis of miRNAs profiling, coupled with proteomics analysis, has been applied in recent studies to explore the paradigm of miRNAs-regulated molecular mechanisms, including osteopetrosis [19], kidney [20], and lung diseases [21]. Such integrative post-transcriptomics analysis provides an understanding of the responses that follow exposure to PM_2.5_. Identifying the miRNAs and proteins involved in these responses would not only provide a better understanding of the underlying mechanisms, but also reveal potential biomarkers of exposure.

miRNAs have been intensively reported to be involved in the development and metastasis processes of lung cancer [22-24]. Hereby, we hypothesized that post-transcriptomics could facilitate the identification of key miRNAs and proteins that played important roles in PM_2.5_-related lung cancer. The miRNAs identified in the present study could be used as a potential index for evaluating PM_2.5_ exposure levels in humans.

## RESULTS

### Overview of miRNA microarray and proteomics analysis

**Figure 1:**
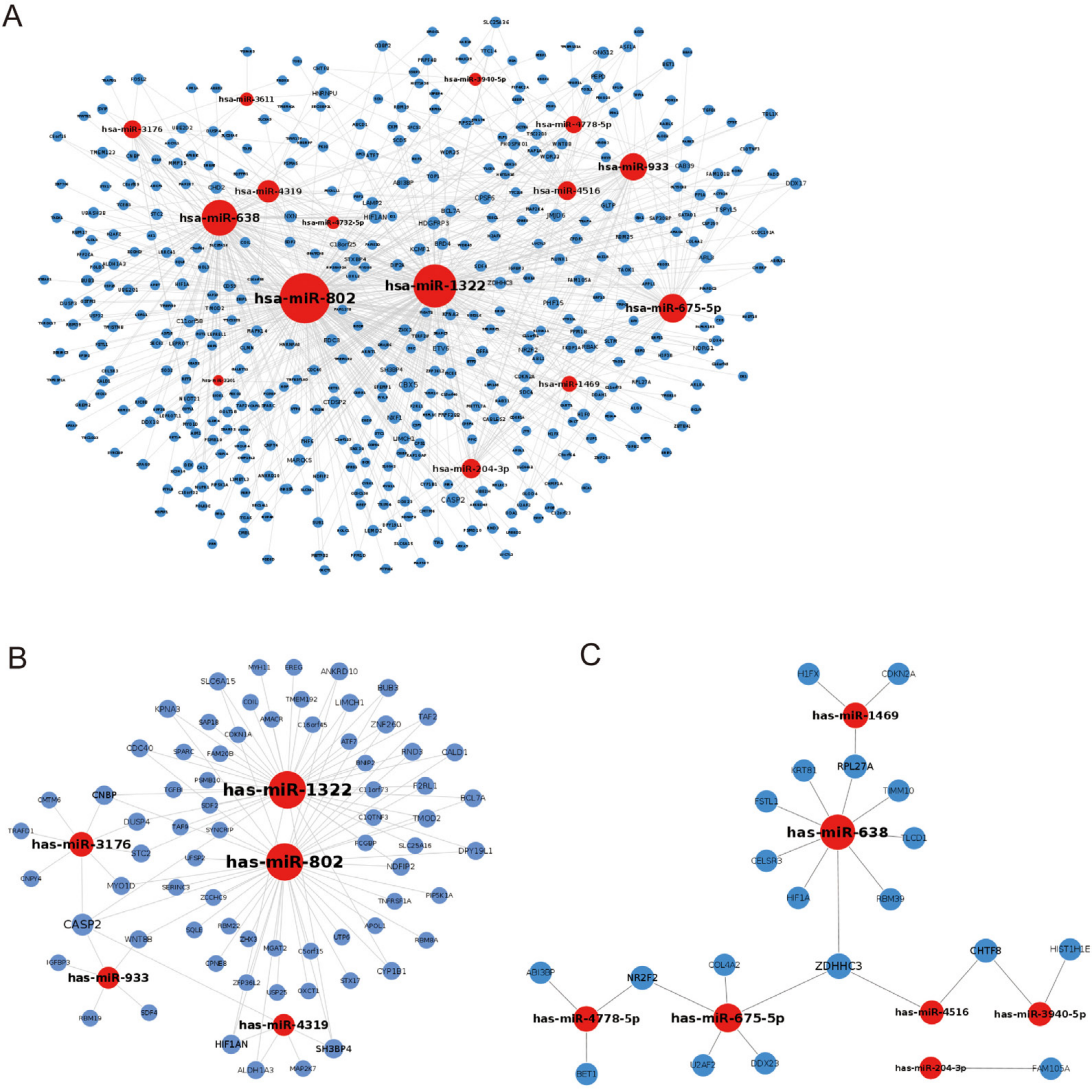
Inhibition of miRNAs to their target protein-encoding genes **A.** The potential targets of differentially expressed miRNAs that were significantly modulated, as determined by miRNA microarray. **B.** Downregulated miRNAs and their potential upregulated target genes. **C.** Upregulated miRNAs and their potential downregulated target protein-encoding genes.

A total of 20 modulated miRNAs from 500 μg/mL PM_2.5_-treated A549 cells (Table 1) were identified according to cut-off, as fold change > 2 and FDR < 0.05, and the potential target mRNAs of miRNAs were predicted by miRWalk. These protein-encoding mRNAs were screened according to proteomic profiling analysis, with a cut-off of 1.5-fold change. The regulations between significantly modulated miRNAs and their target mRNAs are shown in Figure 1A. Downregulated miR-1322, −802, −3176, −933, −4319, and their target mRNA-encoded proteins, which were predicted to be upregulated according to their proteomics profiles, are shown in Figure 1B. Upregulated miR-1469, −638, −4778-5p, −675-5p, −4516, −3940-5p, −204-3p, and their target mRNA-encoded proteins (downregulated) are shown in Figure 1C.

**Table 1:** miRNA microarray predicted modulation of miRNA in PM_2.5_ treated A549 cells

microRNA	Fold change	FDR
hsa-miR-4516	9.687	0.031
hsa-miR-4732-5p	5.867	0.034
hsa-miR-4778-5p	5.563	0.037
hsa-miR-4449	5.352	0.014
hsa-miR-3201	5.278	0.014
hsa-miR-675-5p	4.360	0.037
hsa-miR-638	3.944	0.014
hsa-miR-1469	3.907	0.014
hsa-miR-204-3p	3.212	0.037
hsa-miR-4750-5p	2.967	0.037
hsa-miR-3940-5p	2.848	0.014
hsa-miR-652-5p	2.710	0.041
hsa-miR-3611	2.660	0.022
hsa-miR-802	0.172	0.049
hsa-miR-4319	0.246	0.037
hsa-miR-1322	0.253	0.049
hsa-miR-3176	0.260	0.014
hsa-miR-933	0.260	0.037
hsa-miR-4536-3p	0.317	0.034
hsa-miR-4445-3p	0.345	0.049

### Functional group analysis explored key miRNAs modulation

**Figure 2:**
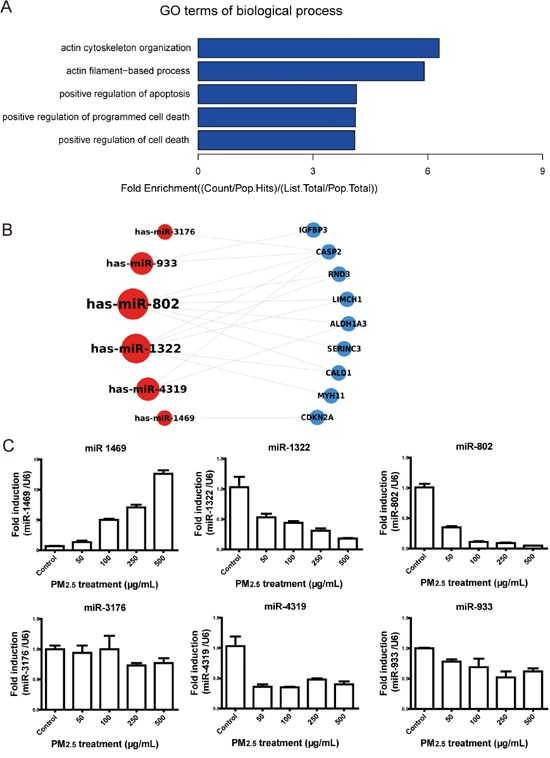
Functional analysis of the potential targets and validation of the significantly modulated miRNAs **A.** The GO enrichment analysis of biological processes showed that most categories were involved in actin-dependent processes and apoptosis. **B.** Nine genes and their related miRNAs were involved in BP enrichment. **C.** The miRNA expression levels were validated by qRT-PCR in A549 cells. The expression of miR-1469, −1322, and −802 were modulated in a dose-dependent manner. ^*^ *P* < 0.05, compared with control.

A total of 91 protein-encoding mRNAs, which are shown in Figure 1B and 1C, were input to perform GO analysis, and the results showed that the significantly enriched terms for GO biological processes were actin and apoptosis-related processes (Figure 2A and Table 2). The miR-802-regulated genes Rnd3, LIMCH1, and CALD1; and the miR-1322-regulated gene MYH11 were associated with actin-dependent processes. SERINC3, CDKN2A, ALDH1A3, IGFBP3, and CASP2 were involved in apoptosis or cell death, and were regulated by miR-802, −4319, −933, −1322, −3176, and miR-1469. The nine mRNAs involved in the GO biological processes and encoding proteins are shown in Figure 2A and Table 3, and their related six miRNAs are shown in Figure 2B. miR-802 and −1322 appeared to be related to both actin- and apoptosis-involved mRNAs. miR-933, −1469, −3176, and −4319 were associated with apoptosis processes.

**Table 2:** Gene ontology enrichment analysis of miRNA target mRNAs

GO Term	P-Value	Genes	Involved miRNAs
actin cytoskeleton organization	0.023554309	RND3, CALD1, LIMCH1, MYH11	miR-802, miR-1322
actin filament-based process	0.027802287	RND3, CALD1, LIMCH1, MYH11	miR-802, miR-1322
positive regulation of apoptosis	0.029075368	SERINC3, CDKN2A, ALDH1A3, IGFBP3, CASP2	miR-802, miR-4319, miR-933, miR-1322, miR-3176, miR-1469
positive regulation of programmed cell death	0.029725847	SERINC3, CDKN2A, ALDH1A3, IGFBP3, CASP2	miR-802, miR-4319, miR-933, miR-1322, miR-3176, miR-1469
positive regulation of cell death	0.030164333	SERINC3, CDKN2A, ALDH1A3, IGFBP3, CASP2	miR-802, miR-4319, miR-933, miR-1322, miR-3176, miR-1469

**Table 3:** Gene ontology enrichment involved mRNAs and functions of their encoding proteins

protein GI	Gene symbol	Protein description	Fold change
153266822	ALDH1A3	aldehyde dehydrogenase family 1 member A3	1.780
15149465	CALD1	caldesmon isoform 5	1.676
39995059	CASP2	caspase-2 isoform 1 preproprotein	1.598
62243068	IGFBP3	insulin-like growth factor-binding protein 3 isoform b precursor	3.671
163310745	LIMCH1	LIM and calponin homology domains-containing protein 1 isoform c	7.925
13124875	MYH11	myosin-11 isoform SM2A	2.869
4885069	RND3	rho-related GTP-binding protein RhoE precursor	1.835
39812106	SERINC3	serine incorporator 3 precursor	1.529
4502749	CDKN2A	cyclin-dependent kinase inhibitor 2A isoform p16INK4a	0.617

To validate the modulation of miRNAs in PM_2.5_-treated A549 cells, the six miRNAs involved in GO enrichment were further validated by qRT-PCR in A549 cells treated with 50, 100, 250, and 500 μg/mL PM_2.5_ (Figure 2C). Consistent with miRNA microarray data, miR-1469 expression was significantly upregulated with 500 μg/mL PM_2.5_ treatment, as compared to the control (*P* < 0.05). The expression of miR-1322, −802, −933, −3176, and −4319 were significantly downregulated at this concentration, as compared to the control (*P* < 0.05). However, only the modulations of miR-1469, −1322, and −802 were in a dose-dependent manner.

### miR-802 mimics rescue against PM_2.5_-induced damages

**Figure 3:**
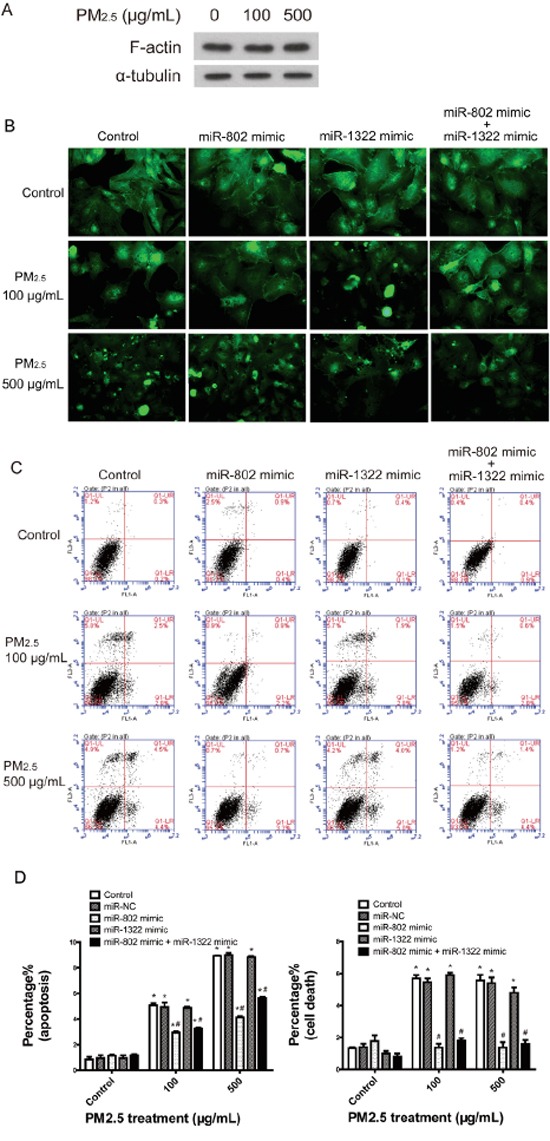
Cell-based assays confirmed the regulation to cellular phenotypes by miR-802 **A.** Immunodetection of F-actin expression in A549 cells. **B.** PM_2.5_ treatment induced A549 cell actin cytoskeletal reorganization and miR-802 mimic partially rescued actin reorganization. **C** and **D.** PM_2.5_ significantly increased the proportion of cells that underwent apoptosis and cell death, and the miR-802 mimic partially protected the cells against apoptosis and fully rescued the cell death phenotype in A549 cells. ^*^P < 0.05, compared with untreated control, ^#^*P* < 0.05, compared with control within each group.

To investigate the PM_2.5_ treatment induced phenotypes of A549 cells according to GO enrichment analysis, we initially evaluated the cytoskeletal organization of actin. The content of actin in A549 cells did not show obvious alterations after PM_2.5_ treatment (Figure 3A), while the microscopic evaluation of actin staining revealed that most A549 cells treated with PM_2.5_ displayed a disorganized and aggregated actin phenotype. The visibly altered actin stress fiber reorganization could be partially or totally rescued by miR-802 mimics with low or high doses of PM_2.5_ (Figure 3B). Apoptosis in a dose-dependent manner and cell death induced by PM_2.5_ were evaluated by flow cytometric analysis. Only miR-802 mimics partially rescued cell death and apoptosis (Figure 3C and 3D). The miR-1322 mimic did not attenuate PM_2.5_-induced actin disorganization and apoptosis.

### miR-802 target genes are involved in cellular phenotypes

**Figure 4:**
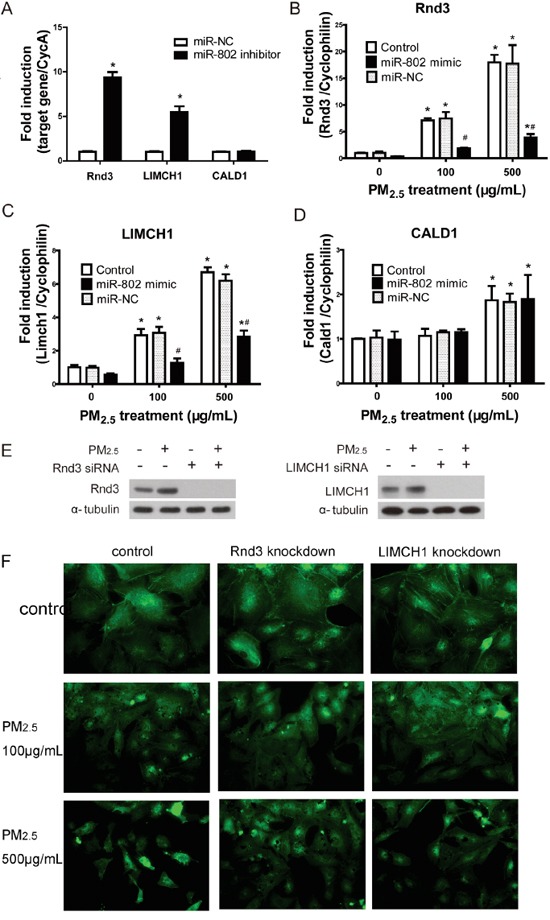
The regulation of miR-802 to Rnd2, LIMCH1, CALD1, and the associated cellular phenotype **A.** The expression of Rnd3 and LIMCH1 increased significantly after the transfection of miR-802 inhibitor. **B.** mRNA expression level of miR-802 target gene increased in A549 cells following PM_2.5_ treatment; after miR-802 mimic transfection, expression of Rnd3 and **C.** LIMCH1 attenuated to control levels. **D.** The expression of CALD1 was not affected by miR-802 mimic. **E.** miR-802 target Rnd3 and LIMCH1 were knocked down in A549 cells, and their protein expression levels were not affected by PM_2.5_ exposure. **F.** After knocking down of Rnd3 or LIMCH1 in A549 cells, actin organization was not regulated by PM_2.5_ treatment. ^*^ *P* < 0.05, compared with untreated control. ^#^ *P* < 0.05, compared with control within each group.

Since miR-802 appeared to be involved with PM_2.5_-induced A549 cell damage, we further assessed the suppression of miR-802 to its target genes. GO analysis suggested that actin-related processes were the most enriched category and that the involved genes, Rnd3, LIMCH1, and CALD1, were potential targets of miR-802. The mRNA expression levels of these genes were evaluated following treatment of 100 or 500 μg/mL PM_2.5_, with or without miR-802 mimics. The increased expression of Rnd3, LIMCH1, and CALD1, following treatment with PM_2.5_, was consistent with the proteomics data and accounted for the upregulation of the encoding proteins. Once the expression of miR-802 was inhibited in A549 cells, enhanced expression levels of Rnd3 and LIMCH1 were exhibited (Figure 4A). The miR-802 mimic ameliorated the mRNA expression levels of Rnd3 and LIMCH1following PM_2.5_ exposure, but not of CALD1 (Figure 4B, 4C and 4D).

To further assess the association between Rnd3, LIMCH1, and PM_2.5_-induced actin disorganization, Rnd3 or LIMCH1 were knocked down in A549 cells. The expression of Rnd3 and LIMCH in A549 cells was not influenced by PM_2.5_ exposure after their encoding genes were knocked down (Figure 4E). As shown in Figure 4F, once Rnd3 or LIMCH1 was knocked down, the disorganization of actin attenuated, as compared to the PM_2.5_-treated groups.

### Alveolitis and bronchial dysplasia were observed in PM_2.5_-treated mice lungs

**Figure 5:**
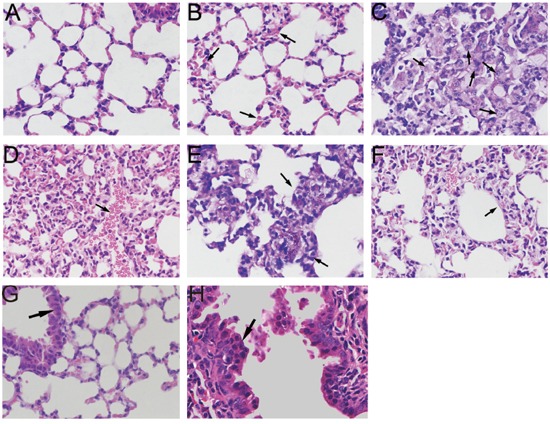
Microscopic examination of mice lung tissues exposed to PM_2.5_ **A.** Light microscopy analysis of the lung sections from filtered clean air-treated mice showed a normal appearance. **B.** Pneumorrhagia was observed in 7 days PM_2.5_-treated mice lungs. **C.** Macrophages located in alveolar spaces with a foamy appearance coupled with pronounced lymphocytic reaction were observed in 7 days PM_2.5_-treated mice lung. **D.** 14 days PM_2.5_-treated mice lung section showed diffuse pneumorrhagia. **E.** Inflammation infiltration surrounding black pigment deposit. **F.** Widening of the inter-alveolar septae were observed in mice lung following 14 days PM_2.5_ treatment. **G.** Normal single layer epithelium with basally located nuclei. **H.** Dysplasia of epithelium, characterized with multi-layer horizontal orientation of nuclei was observed in mice lung tissues exposed to PM_2.5_ for 28 days.

Obvious pathological changes were observed in PM_2.5_-treated lung tissues by microscopic examination. As shown in Figure 5A, lung sections from filtered clean air treated mice had a normal appearance. Interstitial pneumorrhagia was observed in a time-dependent manner in PM_2.5-_exposed animals (Figure 5B, 5D). Enlarged and foamy macrophages, associated with pronounced lymphocyte infiltration, were observed in mice lungs treated with PM_2.5_ for 7 days (Figure 5C). This type of macrophage reaction was clearly adverse and had the potential to induce long-term, irreversible alterations to the pulmonary structure [25]. Massive lymphocytes surrounding the black pigment deposited in lung tissue and thickened alveolar walls were demonstrated in mice lungs after 28 days of treatment (Figure 5E, 5F). Figure 5G showed normal bronchial epithelium, with basally located nuclei and apical cilia. Following 28 days exposure to PM_2.5_, bronchial dysplasia were observed in mice lung tissue, characterized by stratified non-ciliated epithelium and the horizontal orientation of nuclei [26].

**Figure 6:**
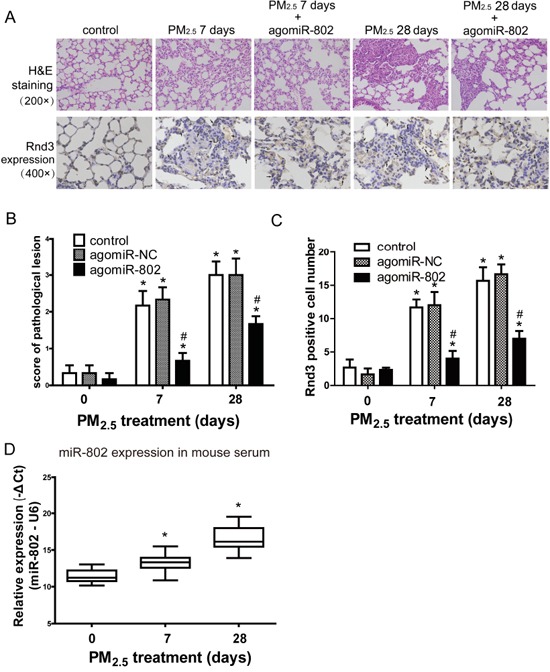
Patholigical lesions and Rnd3 expression in the lungs of mice after PM_2.5_ exposure **A.** Microscopic examination of the pathological lesion and Rnd3 expression in lungs of mice. **B.** Scoring of pathological lesions. **C.** Rnd3 Positive cell numbers in lung tissues. **D.** miR-802 expression was downregulated in animal serum treated with PM_2.5_ in a dose-dependent manner. ^*^ *P* < 0.05, compared with untreated control. ^#^ *P* < 0.05, compared with control within each group.

The pathological lesions score and immunohistochemistry staining were used to evaluate the rescue of agomiR-802 *in vivo* (Figure 6). Expressions of CALD1, Rnd3, and LIMCH1 were examined by immunohistochemistry staining. Only the expression of Rnd3 demonstrated differences between the control and PM_2.5_-treated mice lung tissues (Figure 6A). Sporadic Rnd3-positive cells located in normal alveolar walls and increased Rnd3 expression were observed in the inter-alveolar tissue after 7 days of PM_2.5_ treatment, and the thickening of the alveolar wall was seen after 28 days in PM_2.5_-treated mice lungs. Decreased Rnd3 expression was observed in the lungs of agomiR-802-treated mice.

As shown in Figure 6B, lung tissues exhibited moderate alveolitis in PM_2.5_-treated mice on day 7, with or without agomiR-NC. Severe alveolitis was observed 28 days after PM_2.5_ treatment, with or without agomiR-NC. AgomiR-802 partially rescued PM_2.5_-induced pulmonary damage *in vivo*, of which the score was significantly higher than the control and lower than the corresponding PM_2.5_-treated mice (*P* < 0.05). The similar modulated trend of Rnd3 positive cell numbers were showed in Figure 6C. Since miR-802 is a circulating miRNA [27] and is expressed in both humans and mice, we assessed the expression levels of miR-802 in the serum of PM_2.5_-treated mice. As shown in Figure 6D, miR-802 was significantly downregulated in PM_2.5_-treated mouse serum.

### Long-term exposure to PM_2.5_ enhanced carcinogenesis and metastasis of A549 cells

**Figure 7:**
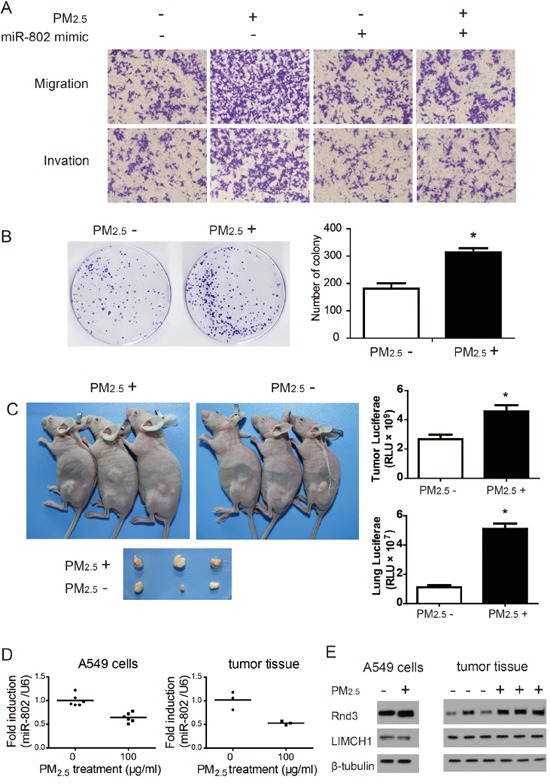
Long-term PM_2.5_ exposure regulates *in vitro* and *in vivo* A549 cell carcinogenesis and metastases **A.** Cell migration and invasion capacity. **B.** Colony formation ability of A549 cells was significantly increased following long-term PM_2.5_ exposure. **C.** Flank tumor and lung metastatic burden measured by luciferase activity. **D.** miR-802 expression levels were down regulated. **E.** Rnd3 expression was enhanced in A549 cells and flank tumor after long-term PM_2.5_ treatment. ^*^ *P* < 0.05, compared with control.

In terms of malignancy, cell motility reflects the invasive capacity of cells, and is associated with tumor metastasis and recurrence [28]. To determine the effects of long-term PM_2.5_ exposure on A549 cells, invasion through Matrigel and migration through Transwells of A549 cells were evaluated. Relative to the control, long-term PM_2.5_ exposure promoted the invasion and migration of A549 cells (Figure 7A). A foci formation assay demonstrated that the colony number of long-term PM_2.5_ treated cells was significantly increased, as compared to the control group (Figure 7B).

Tumorigenesis of A549 cells *in vivo* were then examined by subcutaneous injection to nude mice. Long-term PM_2.5_ exposure increased the tumor sizes relative to the control group (Figure 7C). Meanwhile, the metastatic potential for flank tumors in mice was evaluated through luciferase biochemical measurements. Markedly enhanced luciferase activities in mice flank tumors and lungs were observed in mice derived from stably transformed and long-term PM_2.5_ exposure A549 cells, as shown in Figure 7C, suggesting an increased lung metastatic burden. These data indicated that long-term PM_2.5_ exposure was involved in the neoplastic and metastatic capacity of A549 cells.

Furthermore, the expression levels of miR-802, LIMCH1, and Rnd3 were assessed in A549 cells and mice flank tumor tissues. A significant decrease of miR-802 expression was observed in long-term PM_2.5_ exposure A549 cells and flank tumors, relative to the control groups (Figure 7D). These same patterns were observed with Rnd3 expression levels through immunoblotting assays (Figure 7E).

### Notable enhancement of Rnd3 expression are observed in lung cancer specimens

**Figure 8:**
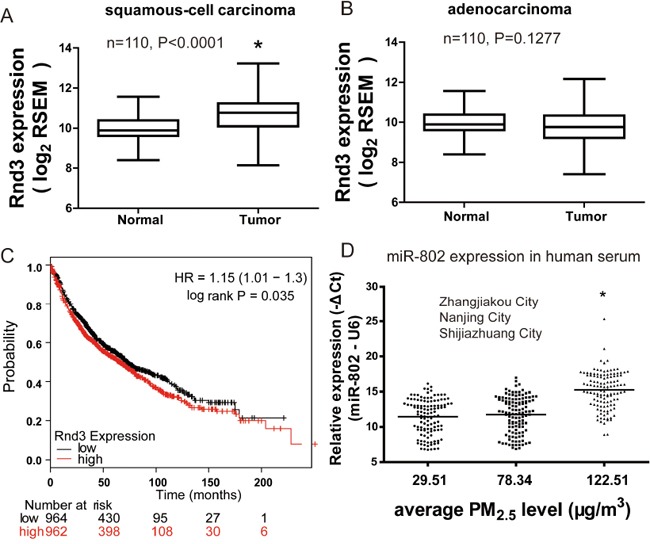
Rnd3 expression is up-modulated and unfavorable prognostic indicator in lung cancer Aberrant expression of Rnd3 in **A.** human squamous cell carcinoma lung cancer or **B.** adenocarcinoma lung cancer and paired normal tissues. Data were retrieved from the TCGA database. **C.** KM plot demonstrated that lung cancer patients with higher Rnd3 expression had shorter survival time. hazard ratio (HR) **D.** miR-802 expression significantly decreased in the serum of residents living in a moderately polluted city. ^*^ *P* < 0.05, compared with control.

Rnd3 mRNA expression in 110 paired human lung cancer specimens and normal tissues were retrieved from the TCGA database. It was found that the median Rnd3 level in lung tumor tissues was significantly higher than that in normal tissues. However, this marked increase only occurred in squamous cell carcinoma, not in adenocarcinoma (Figure 8A and 8B).

To determine the relationship between Rnd3 levels and the prognosis for lung cancer patients, the correlation between Rnd3 expression and overall survival (OS) was evaluated by Kaplan-Meier analysis. The OS for patients with high Rnd3 expression was lower than that of those with low Rnd3 expression (Figure 8C), suggesting that Rnd3 was a prognostic indicator for OS of patients with lung cancers. The expression levels of miR-802 were significantly decreased in human serum samples that were collected from moderately PM_2.5_-polluted (Shijiazhuang), as compared to unpolluted city(Zhangjiakou) (Figure 8D), indicating the potential application of miR-802 as a PM_2.5_ exposure biomarker.

## DISCUSSION

In the present study, we provide novel evidence of the association between miRNAs and their potential target proteins in PM_2.5_-induced lung cancer through integrative analyses of post-transcriptional profiling.

Lung cancer is the most common cancer and the leading cause of cancer death in China in recent years [29]. As a frequent target of injury, alveolar epithelial cells are key elements in the pathobiology of lung diseases. Repeated stimulus or injury, such as persistent air pollution, could superimpose inflammation and hypoxia onto pulmonary tissues and overwhelm repair pathways [30]. Actin cytoskeleton has been implicated in both the location of the mitotic spindles and the formation and remodeling of adherens junctions [8, 31]. In the present study, the omics profiling data, coupled with bioinformatics analysis, strongly indicated the importance of actin-dependent processes in PM_2.5_-induced cellular damage. Inconsistent with the results of Dysart's group [32], there were no clearly enhanced staining of actin filaments in our study; however, the thick and aligned actin filaments were observed in PM_2.5_-treated cells. This difference might be attributed to the different species of cell models and different exposure doses.

Rnd3/RhoE, a small Rho GTPase, plays an important role in the regulation of the actin cytoskeleton [33]. LIMCH1 encoded LIM and calponin homology domain1 is involved in actin binding. Increased mRNA expression levels in A549 cells account for the upregulation of LIMCH1 and Rnd3 following PM_2.5_ treatment. Although the knockdown of Rnd3 and LIMCH1 demonstrated stimulation to actin organization in cell-based assays, only the enhanced expression of Rnd3 was observed in mouse lungs, following PM_2.5_ treatment. Rnd3 is involved in actin organization, as well as the development of cancers. Rnd3 is indispensable to osteoclast migration and bone resorption by regulating actin turnover in podosomes [34]. In the nervous system, Rnd3 mediates disassembly of actin filaments of radial glia, which coordinates cellular behaviors during the generation of neurons by neural stem cells [33]. Overexpression of Rnd3 might serve as an unfavorable prognostic factor in lung cancer patients [35]. In the non-small cell lung cancer tissues, the expression of Rnd3, mRNA, and protein levels were dramatically increased, compared to that of adjacent non-tumoral lung tissues. Moreover, overexpression of Rnd3 was correlated to patients’ smoking history [36]. Together with our study, overexpression of Rnd3 in lung tissue could be induced by environmental stresses. In the present study, enhanced Rnd3 expression, which was increased by blunted miR-802 expression, was involved in actin disorganization of alveolar epithelial cells and accounted for PM_2.5_-induced lung damage. The analysis of Rnd3 levels in human lung specimens from the TCGA database and K-M plot revealed the association between enhanced Rnd3 expression and lung cancers. Coupled with our bioinformatics analysis and cell- and animal-based assays, these results indicated that exposure to PM_2.5_ played a role in the development of lung cancers.

In 2013, the International Agency for Research on Cancer identified particulate matter (PM) from outdoor air pollution as carcinogenic to humans [1]. In our study, dysplasia of bronchial epithelia was observed after exposure to PM_2.5_ for 28 days. Histological alterations in the bronchial epithelium termed dysplasia are precursors to lung cancer [26]. Therefore, our study provided strong evidence that PM_2.5_ exposure exerted risks to human health.

Although the association between PM_2.5_ modulated miRNAs with lung cancers were reported [37], the carcinogenesis and metastasis capacities of chronic PM_2.5_ exposure are still unknown. It is noted that the invasion and migration of A549 cells following long-term PM_2.5_ exposure were significantly enhanced both *in vitro* and *in vivo*. To the best of our knowledge, this is the first report on the effects of chronic PM_2.5_ exposure.

Among six validated miRNAs, miR-802 was down-modulated in a dose-dependent manner and strongly correlated to the PM_2.5_-induced damage of A549 cells. Moreover, consistent with the omics data and cell-based assay, miR-802 was significantly downregulated in mice serum exposed to PM_2.5_ and in the serum of individuals living in a moderately polluted city (Shijiazhuang). Thus, our results indicated that serum miR-802 could be a potential biomarker for PM_2.5_ exposure levels. Our study proposed a different candidate biomarker for PM_2.5_ exposure from study of Liu et al [37], which could be attributed to the different PM_2.5_ samples used.

One limitation of our study is that only A549 cell line were used to explore the pulmonary effects of PM_2.5_. The further study should include other pulmonary cells, such as HBE or HELF cells, which could facilitate to identify the different or common modulated critical pathways induced by PM_2.5_. The other limitation is that miR-802 expression levels was not significantly down-regulated in slight polluted city (Nanjing), this might attribute to the different components of PM_2.5_ or the limited number of subjects. Therefore, further studies are still required. Taken together, these data suggest that exposure to PM_2.5_ may be a public health concern, as it is a risk factor that could contribute to intractable pulmonary disorders. Chronic exposure to PM_2.5_ could increase the carcinogenesis and metastasis capacities of A549 cells. In addition, miRNA plays an important role as a regulator and an exposure indicator in such processes.

## MATERIALS AND METHODS

### Fine particulate matter (PM_2.5_)

Urban particulate matter (PM_2.5_) (SRM 1648a) was purchased from the National Institute of Standards and Technology (NIST), USA. This Standard Reference Material (SRM) is atmospheric particulate matter collected in an urban area. All constituents provided in SRM 1648a were naturally present in the material before processing. The major components of SRM 1648a were introduced by a research group from NIST [38].

### Study subjects

A total of 360 non-smoking, healthy individuals were recruited for the present study, including 120 individuals (60 males (mean age, 46 years; range 30-61 years) and 60 female (mean age, 44 years, range 30-59 years)) from a tourist city, Zhangjiakou, in Northern China; 120 individuals (60 males (mean age, 45 years; range 32-60 years) and 60 female (mean age, 44 years, range 30-59 years)) from an industrial city, Nanjing, in Eastern China; and 120 individuals (60 males (mean age, 46 years; range 30-60 years) and 60 females (mean age, 42 years; range 30-60 years)) from an industrial city, Shijiazhuang, in Northern China. These individuals were free of cancer, cardiovascular disease, and pulmonary disease, and all of them had lived in their current cities for at least 1 year. We obtained whole blood samples for miRNA analysis from November 2014 to February 2015, with informed consent and agreement. Serum was separated from blood within 1 h by centrifugation at 3,000 g for 10 min, followed by a 15 min high-speed centrifugation at 12,000 g to completely remove the cellular debris. The supernatant serum was collected, as well as the other half volume of whole blood, and both were stored at −80°C until subsequent use. The ethics review board of Southeast University (NO. 2014070012) approved the research protocol, and all of the samples were used in compliance with corresponding ethical regulations.

According to emission inventory data from the Bureau of Meteorology of China, the air pollution index (API) and PM_2.5_ levels during winter (Dec. 2014 to Feb. 2015) were 56.21 and 29.51 μg/m^3^, respectively, in Zhangjiakou city; were 167.54 and 78.34 μg/m^3^, respectively, in Nanjing city; and were 213.44 and 122.51 μg/m^3^, respectively, in Shijiazhuang city. The air pollution levels in Zhangjiakou, Nanjing, and Shijiazhuang were in compliance with the API standards of good, lightly polluted and moderately polluted areas, respectively.

### Cell lines and RNA extraction

The human lung adenocarcinoma cell line A549 (American Type Culture Collection) was maintained in Dulbecco's Modified Eagle's Medium (DMEM), and supplemented with 10% (v/v) fetal bovine serum (FBS) at 37°C in 5% CO_2_. Cells were seeded in 10 cm culture dishes, and exposed to 500 μg/mL urban PM_2.5_ (NIST SRM 1648a, USA), with three biological replicates. Control groups were treated with culture medium. Total RNA was extracted using the TRIZOL reagent (Invitrogen, USA), according to the manufacturer's instructions, 24 h after treatment.

### miRNA microarray analysis

The microarray analysis for miRNA profiling was conducted by the miRCURY LNA Array system (Exiqon, Vedbaek, Denmark). Total RNA was extracted and purified, using the mirVana miRNA Isolation Kit (Ambion, Austin, TX, USA), following the manufacturer's instructions. Each array was hybridized with either Hy3 or Hy5-labeled RNA, using the miRNA Complete Labeling and Hyb Kit (Exiqon) in a hybridization oven. After hybridization, each array was washed and scanned and the raw data were subjected to background subtraction and normalization with the *limma* R-package [39]. Triplicate miRNA probes were averaged and their intensities (>=30) in all samples were saved for calculating normalization factor using the Median normalization method. Discriminant miRNAs and differences between groups were analyzed using Bayes moderated *t* test (limma), with Benjamini Hochberg false discovery rate (FDR) at *P* < 0.05, unless otherwise specified. A cut-off of 2-fold change and FDR<0.05 was applied to select up- and down-regulated miRNAs.

### Proteomics analysis

A549 cells were exposed to 500 μg/mL urban PM_2.5_ with three biological replicates for 24 h, lysed in RIPA buffer, containing protease inhibitor cocktail (Roche, Germany), and incubated on ice for 30 min. Extracted proteins were subsequently tagged with tandem mass tags for quantitative mass spectrometry (TMT^®^ Mass Tagging Kit, Thermo Scientific, Germany) [40]. Liquid chromatography-mass spectrometry (LC-MS/MS) raw data were assessed using Sequest-HT (Thermo Fisher Scientific Lafayette, CO, USA) as a search engine within the Proteome Discoverer version 1.4 against the Human RefSeq database (71465 proteins, updated on 03/03/2014). The results were filtered using the following settings: only high-confidence peptides with a global FDR < 1% based on a target-decoy approach were included. In the TMT quantitation workflow, the most confident centroid method was used with an integration window of 20 ppm. Only unique peptides were used for protein quantification.

### Functional group analysis

Significantly modulated miRNAs were ranked according to the *P* value. The possible binding sites of the top 20 miRNAs were predicted by the online database miRWalk [41]. The predicted mRNA, whose encoding proteins had a fold change greater than 1.5, according to proteomics analysis, was further analyzed using a functional annotation tool, Database for Annotation, Visualization, and Integrated Discovery (DAVID 6.7). The *P* value was set to 0.05 to denote the significance of gene ontology (GO) enrichment in the differentially expressed mRNA list. Fold enrichment ([Count/Pop.Hits/List.Total/Pop.Total]) was used to denote the enrichment of a particular GO term.

### qRT-PCR assays for miRNAs

A549 cells seeded in 10 cm culture dishes were treated with 50, 100, 250, or 500 μg/mL PM_2.5_ for 24 h. Total RNA was isolated from cell lysates, according to the instructions provided by the manufacturer of TRIZOL (Invitrogen, USA). The total RNA from animal and human serum was extracted using Trizol reagent (Invitrogen, USA) and the miRNeasy Serum/Plasma Kit (Qiagen, Germany), respectively.

A total of 1 μg RNA was converted to cDNA using a One Step PrimeScript^®^ miRNA cDNA Synthesis Kit (Takara, Shiga, Japan). The expression levels of miR-802, −1322, −1469, −4319, −933, and −3176 were amplified with PCR primers (RiboBio, China) on a Quant Studio 6 Flex system (Applied Biosystems, Life Technologies, USA). The relative expression levels of miRNAs were normalized against U6, and were calculated using the 2^-ΔΔCt^ method. All of the experiments were performed in triplicates.

### F-Actin staining

F-actin in A549 cells was stained using the Actin-Tracker Green Kit (Beyotime, China), according to the manufacturer's protocol. Briefly, A549 cells were seeded in a 6-well plate and treated with PBS, 100 μg/mL or 500 μg/mL PM_2.5_, with or without miR-802 mimic, miR-1322 mimic, miR-802 and miR-1322 mimic rescues for 24h in 6-well plates, and then fixed in 4% PFA for 10 min. The Actin-Tracker Green was diluted by 1:80 with 0.1% Triton X-100/1% BSA/PBS, and 200 μL working solution was applied to each well for 20 min at room temperature. After washing with 0.1% Triton X-100/PBS three times, the actin morphology was observed under a fluorescent microscope (Olympus, Japan).

### miR-802 inhibitor transfection and validation of miR-802 target gene expression in A549 cells

The miR-802 inhibitor were synthesized by RiboBio Corporation (China). A total of 2 ×10^5^ A549 cells, miR-802 inhibitor with a final concentration of 300 nM and lipofectamine RNAiMAX were seeded in a 6-well plate with triplicates. Control groups were treated with 300 nM miR-NC. Transfection efficiency was detected by RT-qPCR after incubation for 48 h. Total RNA were extracted with TRIZOL.

A549 cells were seeded in 6-well plates at a density of approximately 1 × 10^6^ cells per well, and exposed to 100 or 500 μg/mL PM_2.5_ or control medium for 24 h. To verify the rescue of miR-802 mimic, miR-802 mimic and negative control (miR-NC) were synthesized by RiboBio Corporation (China), followed by transfection using Lipofectamine RNAiMAX (Life Technologies, USA), according to the manufacturer's instructions. Cells were transfected with 40 nM miR-802 or miR-NC, and exposed to 100 or 500 μg/mL PM_2.5_ or control medium for 24 h, after which they were trypsinized and collected. Total RNA was extracted using TRIZOL, according to the manufacturer's protocol.

qRT-PCR assays were performed, as described by Li et al [42]. The primer sequences (forward and reverse) are as follows: CALD1, 5′TGGAGGTGAATGCCCAGAAC3′, 5′GAAGGCGTTTTTGGCGTCTTT3′; LIMCH1, 5′CAGACGCCTTCACCAGATGT3′, 5′GATGAGGCAAGTCGGATTCAG3′; Rnd3, 5′GCTCCATGTCTTCGCCAAG3′, 5′AAAACTGGCCGTGTAATTCTCA3′; Cyclophilin A, 5′CCCACCGTGTTCTTCGACATT3′, 5′GGACCCGTATGCTTTAGGATGA3′. All of the experiments were performed in triplicates. The mRNA levels were relative to cyclophilin A for the indicated genes.

### siRNA knockdown of target genes in A549 cells and immunodetection

For siRNA knockdown, we transfected A549 cells in a 6-well plate with siRNAs (Thermo Fisher Scientific, USA) directed against control, Rnd3, or LIMCH1, using DharmaFECT1 (Thermo Fisher Scientific, USA). After siRNA transfection, cells were incubated for an additional 24 h with PM_2.5_ treatment, followed by harvesting. Proteins were analyzed by immunoblotting with primary antibodies for the following antigens: human LIMCH1 (1:500 dilution; Abcam), Rnd3 (1:1000 dilution; Abcam, USA), or α-tubulin (1:10 000 dilution; Sigma, USA).

A549 cells were treated with 100 or 500 μg/ml PM_2.5_ for 24 h, then the proteins were harvested and analyzed by immunoblotting with the expression of F-actin (1:1000 dilution, Abcam, USA) and α-tubulin (1:10 000 dilution; Sigma, USA).

### Animal treatment

A total of 54 male C57BL/6 mice (20–22 g) were purchased from Shanghai SLRC Laboratory Animal Co. Ltd. (China). Mice were maintained and used according to the guidelines of the Committee on Animal Use and Care of Southeast University. The dynamic inhalation exposure chamber was outfitted with extensive air quality monitoring equipment and an aerosol generator. Mice were divided into nine groups (six mice per group) as follows: control, control with agomiR-802, control with agomiR-NC; mice exposed to PM_2.5_ for 7 days, mice exposed to PM_2.5_ for 7 days with agomiR-802, mice exposed to PM_2.5_ for 7 days with agomiR-NC; mice exposed to PM_2.5_ for 28 days, mice exposed to PM_2.5_ for 28 days injected with agomiR-802, and mice exposed to PM_2.5_ for 28 days with agomiR-NC. Mice were housed six per cage on corncob bedding with *ad libitum* access to food and water. Exposure was performed in two stainless steel whole-body inhalation chambers. One chamber received PM_2.5_, and the other received HEPA-filtered clean air at the same flow rates as the experimental groups. Mice were exposed in each chamber for 6 h per day, from 9 a.m. to 3 p.m. and the mean concentrations of PM_2.5_ were 0.3 mg/m^3^. Light cycles were set on a 12/12 h light/dark cycle. The temperature in the chambers was set to 22.5°C. The cholesterol-conjugated miR-802 mimics and negative controls (agomiR-802 and agomiR-NC, respectively) (Ribobio, China) were injected via the tail vein on treatment days 0, 7, 14 and 21 at a dose of 4 mg/kg.

### Histopathological analysis of mice lung tissues

Mice were euthanized under ether anesthesia 1 h after the end of PM_2.5_ exposure on the seventh or 28^th^ day. Blood was collected from the angular vein, and serum was separated from the venous blood within 1 h by centrifugation at 3,000 g for 10 min, followed by a 15 min high-speed centrifugation at 12,000 g to completely remove cellular debris. The supernatant serum was collected and stored at −80°C until use. All mice were decaptitated on an iced table. One piece of left lung tissue was preserved in 4% paraformaldehyde (PFA) for 24 h at 4°C, embedded in paraffin, serially sectioned (5 μm) and mounted on silane-covered slides. After dewaxing, sections from each mouse were stained with hematoxylin and eosin (H&E) and evaluated under a light microscope to examine the histology of the lung tissues.

The severity of pathological changes was scored according to Szapiel's method [43], with minor modifications: 0 = no apparaent alveolitis; 1 = mild alveolitis with inflammatory cell infiltration and alveolar septum thickening, with only local lesions or those limited to the subpleural areas that did not exceed 20% of the lung; 2 = moderate alveolitis with the involved area more than 20% but less than 50% of the lung; 3 = severe alveolitis with lesions more than 50% but less than 75% of the lung and consolidation changes; and 4=severe alveolitis with lesions more than 75% of the lung, with inflammatory cells inside the alveolar cavity and consolidation changes.

### Immunohistochemistry staining

After dewaxing, immunohistochemistry staining were performed as description [42], and incubated overnight at 4°C with mouse monoclonal antibodies against Rnd3 (1:100) (Thermo Scientific, USA), CALD1 (1:200) (Abcam, USA), and LIMCH1 (1:100) (Abcam, USA). Antibody binding to tissue sections was visualized with a biotinylated rabbit anti-mouse IgG antibody (1:400; DAKO) and developed using diaminobenzidine (DAB) as a substrate. For the negative controls, the primary antibodies were omitted. Each section was examined under microscopy by two histologists. The number of Rnd3 positive cells of each section was counted in three non-overlapping high-power fields (×400 magnification) and analyzed.

### Expression plasmid construction and Lentivirus stable transduction

We generated lentiviral (LTV) expression shuttle vectors (pLenti6/V5-GW/lacZ, Invitrogen) that harbored a firefly luciferase cDNA. Lentiviruses were generated by co-transfection with packaging plasmids pSPAX2 and pMD2G. Lentiviral particles were mixed gently, and added to the A549 cells. After gently swirling to mix, cells were incubated overnight. After 12 hr, culture medium was replaced with 2 ml of complete medium containing 10 μg/ml blasticidin S, which was replaced every 2 days until 1 week after all the control cells had died. Positive cells were maintained in 1 μg/ml blasticidin S for 2 weeks, and then were frozen down until use. For experiments, cells were thawed and allowed to grow for three passages before use.

### Long-term treatment of A549 cells with PM_2.5_ and immunodetection

For long-term exposure, 1 ×10^6^ cells were seeded into 10-cm (diameter) dishes for 24 h and maintained in 0 (control) or 100 μg/ml PM_2.5_ for 48-72 h per passage. This process was continued for 30 passages.

Total RNA of control or long-term PM_2.5_ treated A549 cells was isolated from tumor lysates according to the instructions provided by the manufacturer of TRIZOL. The expression levels of miR-802 in cells were then detected, as in the previous description.

Control or long-term PM_2.5_ treated A549 cells were harvested for immunodetection. Proteins were analyzed by immunoblotting with primary antibodies LIMCH1 (1:500 dilution; Abcam), Rnd3 (1:1000 dilution; Abcam, USA) or α-tubulin (1:10 000 dilution; Sigma, USA).

### Cell migration and cell invasion assays

For cell migration assays, 1 × 10^5^ control or long-term PM_2.5_ treated A549 cells were transferred into a Transwell insert, and incubated with complete medium for 24 h. Cell migration was detected from triplicates for each treatment after crystal violet staining. For cell invasion assays, 1 × 10^5^ control or long-term PM_2.5_ treated A549 cells were transferred into the Transwell insert containing wells pre-filled with Matrigel (CytoSelect 24-Well Cell Invasion Assay Kit; Cell Biolabs, USA) and cultured with complete medium for 48 h. Cell invasion was determined from triplicates for each treatment after crystal violet staining.

### Colony formation analysis

Five hundred control or long-term PM_2.5_ treated A549 cells were seeded in triplicate 10 cm plates and allowed to attach for 24 h. After 24 h, cells were treated with complete media for 10 days. Media was not changed throughout the experiment. Colonies were then stained with 1% crystal violet in ethanol/PBS (15%/85%). Cells were imaged and colony number determined using ImageJ software.

### *In vivo* and *ex vivo* nude mice flank tumor experiments

Mice were maintained and used according to the guidelines of the Committee on Animal Use and Care of Southeast University. Female mice were used for control or long-term PM_2.5_ treated A549 cell implantation.

Mice were injected subcutaneously on the left dorsal flanks, with 5×10^6^ luciferase-expressing stably transformed A549 cells suspended in 0.5 ml DMEM. Fourteen days after injection, mice were killed under ether anesthesia. Lung, liver, and tumor tissues were removed for biochemical luciferase activity analysis. The activities of luciferase were determined by a luminometer (Sirius, Berthold Detection Systems, Germany).

Total RNA of tumors was isolated from tumor lysates, according to the instructions provided by the manufacturer of TRIZOL (Invitrogen, USA). Then the expression levels of miR-802 in tumor tissues were detected, as in the previous description.

Protein levels of tumors were analyzed by immunoblotting with primary antibodies LIMCH1 (1:500 dilution; Abcam), Rnd3 (1:1000 dilution; Abcam, USA) or α-tubulin (1:10 000 dilution; Sigma, USA).

### Retrieval of data from online databases

RNA expression levels of *Rnd3* in lung cancer and their paired normal tissues were obtained from The Cancer Genome Atlas database (TCGA, http://cancergenome.nih.gov/). The Kaplan-Meier survival plot of Rnd3 expression in lung cancer cases was drawn by an online tool of Kaplan Meier-plotter (http://kmplot.com.analysis/) [44], including 2437 lung cancer cases and 2435 controls.

### Data analysis

Values of cell viability, apoptosis and mitochondrial dysfunction assays were expressed as mean ± standard error of the mean (SEM). Statistically significant differences were determined by one-way ANOVA, followed by Dunnett's multiple comparison tests for apoptosis, cell death and Rnd3 expression in lung tissues. The Kruskal-Vallis test was used for the ranked data of pathology score analysis. The methods of *t*-test was used to compare the results of colony formation, TCGA retrieved data, miR-802 expression in A549 and tumor tissues after long-term PM_2.5_ exposure. The method of 2^−ΔΔCt^ was used to analyze the qRT-PCR results in cellular experiments. The method of −ΔCt was used to express the results of qRT-PCR in mouse and individual serum samples. Statistical analysis was performed using SPSS 12.0, and the significance was set at *P* < 0.05
